# Correction: Materials with low dielectric constant and loss and good thermal properties prepared by introducing perfluorononenyl pendant groups onto poly(ether ether ketone)

**DOI:** 10.1039/c8ra90063a

**Published:** 2018-07-30

**Authors:** Handong Sun, Yunxia Lv, Chongyang Zhang, Xiaodan Zuo, Mengzhu Li, Xigui Yue, Zhenhua Jiang

**Affiliations:** Key Laboratory of High Performance Polymers of the Ministry Education of China, College of Chemistry, Jilin University 2699 Qianjin Street Changchun 130012 People’s Republic of China 35192993@qq.com; National Center of Quality Supervision and Inspection of Automobile Spare Parts 6888 Nanhu Road Changchun 130012 China yuexigui@jlu.edu.cn +86-431-85168868

## Abstract

Correction for ‘Materials with low dielectric constant and loss and good thermal properties prepared by introducing perfluorononenyl pendant groups onto poly(ether ether ketone)’ by Handong Sun *et al.*, *RSC Adv.*, 2018, **8**, 7753–7760.

The authors regret that in the published paper, the affiliations of the authors were wrongly designated. The corrected version is shown here.

The Royal Society of Chemistry apologises for these errors and any consequent inconvenience to authors and readers.

## Supplementary Material

